# Case report: Cilioretinal artery occlusion combined with central retinal vein occlusion as the initial presentation of pulmonary arterial hypertension

**DOI:** 10.3389/fmed.2024.1493834

**Published:** 2024-12-20

**Authors:** Yane Gao, Xiaofeng Xie, Jiao Li, Qingshuai Mu, Xingrong Wang, Shuya Wang

**Affiliations:** ^1^Department of Ophthalmology, Affiliated Eye Hospital of Shandong University of Traditional Chinese Medicine, Jinan, China; ^2^Department of Ophthalmology, Shandong Provincial Key Laboratory of Integrated Traditional Chinese and Western Medicine for Prevention and Therapy of Ocular Diseases Shandong Academy of Eye Disease Prevention and Therapy, Jinan, China; ^3^Department of Ophthalmology, Shandong University of Traditional Chinese Medicine, Jinan, China

**Keywords:** pulmonary arterial hypertension, central retinal vein occlusion, cilioretinal artery occlusion, ventricular septal defect, ocular circulation, hemodynamic changes

## Abstract

**Background:**

Pulmonary arterial hypertension (PAH) is characterized by elevated pulmonary artery pressure and vascular resistance, leading to systemic venous hypertension and potential right heart failure. These elevated pressures can extend to ocular veins, resulting in complications such as central retinal vein occlusion (CRVO). This case report highlights a rare instance of CRVO combined with cilioretinal artery occlusion (CilRAO), an uncommon ocular manifestation associated with PAH.

**Case presentation:**

A 13-year-old girl with a history of surgically repaired ventricular septal defect presented with sudden vision loss and a central visual field defect. Investigation confirmed CRVO and an unusual concurrent CilRAO. Although laboratory tests were inconclusive, the echocardiographic examination suggested severe pulmonary arterial hypertension (PAH). The patient received treatment with Bosentan and traditional Chinese medicine, which improved her vision to 20/20, though a paracentral scotoma remained.

**Conclusion:**

This case underscores the critical link between systemic cardiovascular abnormalities and ocular health in PAH, where elevated venous pressure can result in severe and distinctive ocular manifestations. The co-occurrence of CRVO and CilRAO in this patient highlights the susceptibility of cilioretinal arteries to hemodynamic changes, as these arteries lack autoregulatory capacity. Additionally, this case demonstrates the potential for positive outcomes in ocular lesions with targeted PAH therapy. Importantly, it emphasizes the need for vigilance when managing complex cases where conclusions cannot be drawn solely from ocular findings. A multidisciplinary approach and comprehensive diagnostics are essential for identifying underlying causes, ensuring active treatment, and preventing permanent vision loss and serious systemic complications.

## Introduction

Pulmonary arterial hypertension (PAH) is characterized by elevated pulmonary artery pressure and vascular resistance, leading to right heart failure and increased systemic venous pressure (1). This elevated pressure can extend to the cavernous sinus and ophthalmic veins, resulting in ocular complications such as central retinal vein occlusion (CRVO). Cilioretinal artery occlusion (CilRAO) is particularly intriguing pathophysiologically because it receives blood supply from the choroidal circulation rather than the retinal circulation (2). The combination of CilRAO and CRVO presents a unique clinical scenario that warrants thorough examination and discussion. This case report presents a novel instance of PAH-related CRVO leading to CilRAO, underscoring the intricate relationship between systemic cardiovascular conditions and ocular health.

## Case report

A 13-year-old girl was referred to our hospital in April 2023 with sudden, painless vision loss and a central visual field defect in her left eye persisting for 3 weeks. Her medical history included a surgically repaired ventricular septal defect (VSD) at age eight. She reported no significant personal or family medical history, and no prior use of medication.

**Figure 1:**
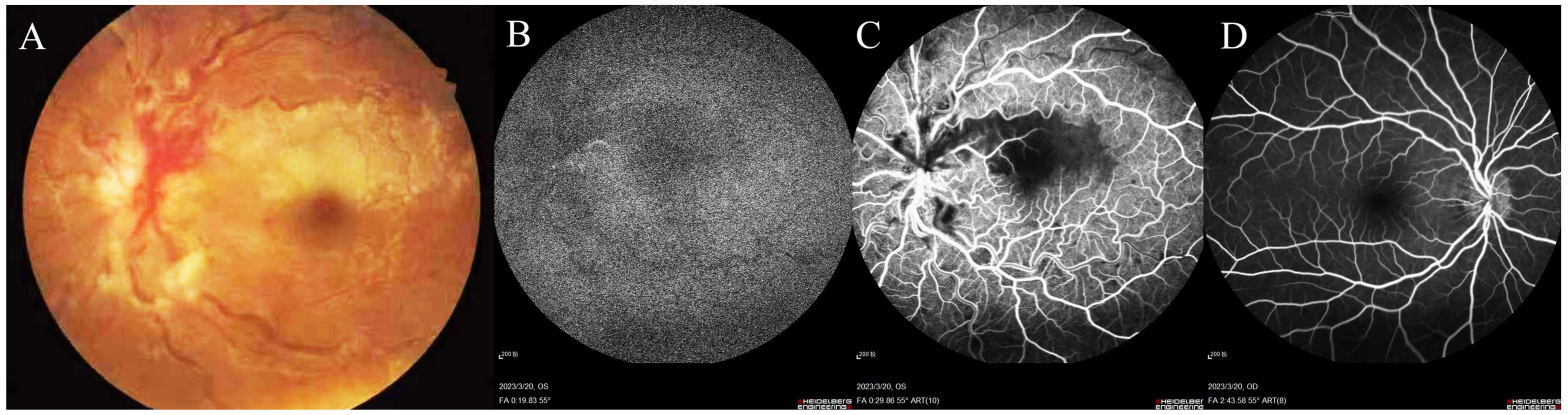
Fundus photography of the left eye reveals pale gray-white retinal swelling above the fovea along the superior papillomacular bundle, with a blurred optic disc margin, flame-shaped hemorrhages, and prominent dilation and tortuosity of retinal veins **(A)**. FFA demonstrates delayed background fluorescence and cilioretinal artery perfusion **(B)**. Hypofluorescence extends from the fovea to the superior vascular arcade, with dilated, tortuous veins showing delayed venous filling **(C)**. The right eye shows mild vessel tortuosity without optic disc stasis or fluorescein leakage, indicative of pseudopapilledema **(D)**.

Two weeks prior, an evaluation at a local hospital recorded a best-corrected visual acuity (BCVA) of 20/20 in the right eye and 20/50 in the left eye, with normal intraocular pressures (IOP) bilaterally. Fundus photography revealed pale gray-white retinal swelling above the fovea along the superior papillomacular bundle, flame-shaped hemorrhages, and blurred optic disc margins in the left eye, accompanied by significant retinal vein dilation and tortuosity (Figure 1A). The right eye showed mild disc edema but was otherwise normal. Fluorescein angiography (FFA) locally showed several notable findings: delayed background fluorescence, delayed perfusion of the cilioretinal artery (Figure 1B), hypofluorescence extending from the fovea to the superior vascular arcade in the posterior pole, as well as dilation and tortuosity of the veins with delayed venous filling (Figure 1C). The right eye showed no evidence of stasis or fluorescein leakage on the optic disc, consistent with pseudopapilledema (Figure 1D).

**Figure 2:**
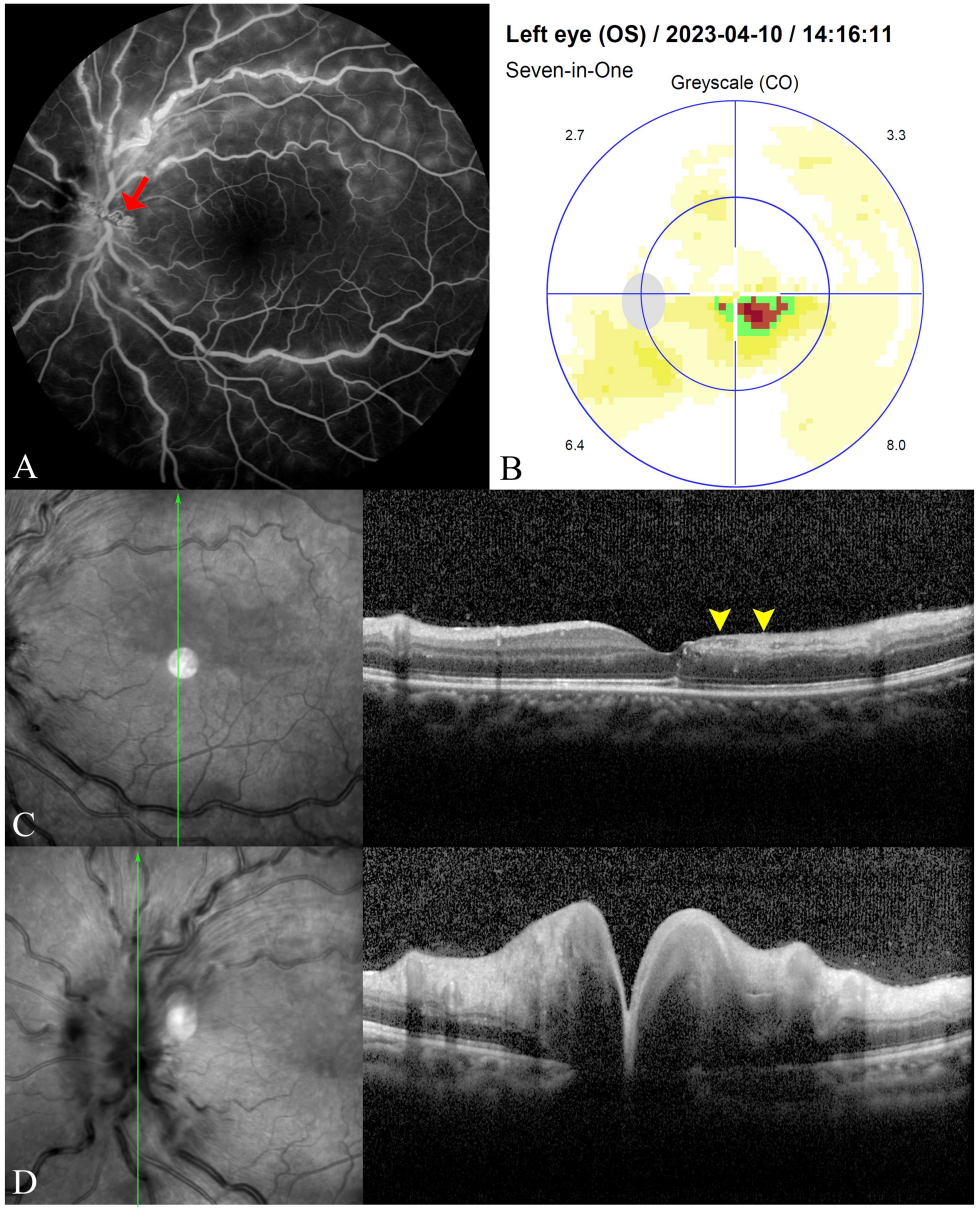
FFA reveals prolonged choroidal perfusion and cilioretinal artery filling time, blockage of fluorescence in areas with scattered intraretinal hemorrhages, widespread capillary dilation and staining, and contrast stasis (red arrow) on the optic disc in the late phase **(A)**. The static visual field test shows a sectoral scotoma adjacent to the fovea in the left eye **(B)**. SD-OCT displays disorganization of the inner macular layers corresponding to hyporeflective areas on NIR imaging (yellow arrowheads), with interspersed hyperreflective foci **(C)**, along with increased thickness and elevation of the optic disc, indicating optic disc edema **(D)**.

Upon admission, her BCVA was 20/50 in the left eye and 20/20 in the right. The ocular adnexa, anterior segment, and IOP were normal, as were pupillary light reflexes. Compared to prior evaluations at the local hospital, fundoscopic examination showed a reduction in the gray-white retinal edema above the fovea in the left eye, with no significant changes otherwise. FFA showed prolonged choroidal perfusion, delayed cilioretinal artery filling, scattered fluorescence blockage, widespread capillary dilation, and optic disc stasis in the late angiographic phase (Figure 2A). The static visual field test recorded a sectoral scotoma adjacent to the fovea in the left eye (Figure 2B). Spectral-domain optical coherence tomography (SD-OCT) revealed disorganization of the inner macular layers, corresponding to hyporeflective areas on near-infrared (NIR) imaging, with interspersed hyperreflective foci (Figure 2C). Optic disc edema was confirmed with increased disc thickness and elevation (Figure 2D). Based on the patient’s symptoms, clinical signs, and comprehensive ophthalmological examination, she was diagnosed with CilRAO combined with CRVO in the left eye.

Laboratory tests revealed mild anemia (hemoglobin: 92 g/L) and a slight elevation in platelet count (385 × 10^9/L). Other tests, including random blood sugar, serum C-reactive protein (CRP), erythrocyte sedimentation rate (ESR), renal function, coagulation profile, antiphospholipid antibodies, homocysteine levels, lipid profile, and D-dimer, were all within normal ranges.

The etiology of CilRAO and CRVO was unclear based on initial findings. Given her VSD history, further investigations were recommended. Despite initial hesitation, the patient, who denied any symptoms beyond ocular complaints and requested immediate ophthalmic treatment, eventually agreed after counseling. Cardiothoracic consultation revealed mild enlargement of the right ventricle and right atrium on echocardiography. The atrial septum appeared intact, with an echogenic patch on the ventricular septum, confirming no shunt signal. Mild dilation of the pulmonary artery and a regurgitant jet in the right ventricular outflow tract were also noted. Tricuspid regurgitation was widely distributed within the right atrium during systole, with a continuous wave (CW) Doppler measurement estimating the pulmonary artery systolic pressure (PASP) at 80 mmHg. Duplex ultrasound of the carotid arteries showed smooth intimal surfaces bilaterally. A Transcranial Doppler (TCD) foaming test detected three emboli in the middle cerebral artery during a Valsalva maneuver, suggesting a potential right-to-left shunt. Head computed tomography (CT) and computed tomography angiography (CTA) of the cerebral and aortic arteries revealed no significant abnormalities.

The patient was diagnosed with severe pulmonary arterial hypertension (PAH) during a cardiothoracic consultation and was prescribed Bosentan at a dose of 31.25 mg twice daily. She also received traditional Chinese herbal treatment to enhance ocular circulation. The decoction included *Prunus persica* (peach kernel), *Carthamus tinctorius* (safflower), Rehmannia glutinosa (raw Rehmannia root), Angelica sinensis (Dong Quai), *Paeonia lactiflora* (red peony root), Ligusticum chuanqiong (Szechuan lovage), and *Achyranthes bidentata* (ox knee), which are traditionally used to promote blood circulation and alleviate stasis. After 1 week of treatment, she requested discharge.

At the one-month follow-up, the patient’s BCVA in the left eye improved to 20/32. Fundus photography showed reduced optic disc edema, venous tortuosity, and intraretinal hemorrhages. OCT revealed asymmetric thinning of the inner retinal layers in the macular region affected by CilRAO, along with substantial improvement in optic disc edema. Echocardiography showed a decrease in PASP to 65 mmHg. The cardiothoracic surgeon advised continuing oral Bosentan therapy.

**Figure 3:**
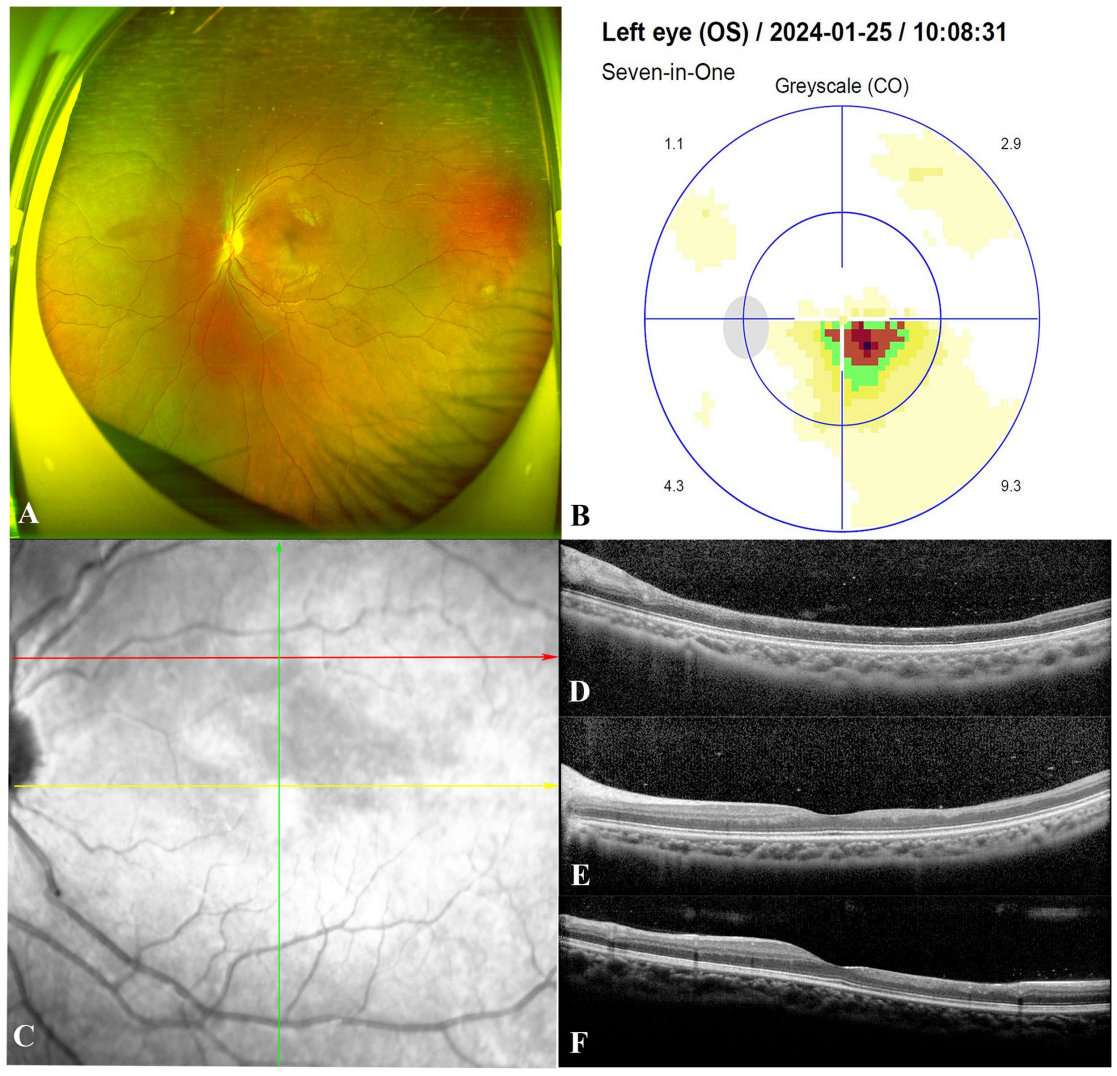
At the follow-up visit 9 months after starting Bosentan treatment. Fundoscopic examination of the patient demonstrated a return to near-normal conditions in the left eye **(A)**. Visual field testing detected a persistent sectoral scotoma adjacent to the macular fovea **(B)**. The red, yellow and green arrows indicate the location and direction of the Spectral-domain optical coherence tomography B-scans in **(D–F)**, respectively. **(C–F)** Illustrated significant thinning of the neural retinal layer in the macular area corresponding to the region of cilioretinal artery occlusion.

At 9 months, the patient’s left eye BCVA improved to 20/20. Fundoscopic examination revealed near-normal conditions (Figure 3A). However, visual field testing detected a persistent sectoral scotoma adjacent to the macular fovea (Figure 3B). OCT showed significant thinning of the inner retinal layers extending temporally and superiorly to the macular fovea (Figures 3C–F), along with complete resolution of optic disc edema. Echocardiography indicated a PASP of 52 mmHg. Liver and kidney function tests were normal, and the patient continued oral Bosentan therapy. At the most recent follow-up, the patient’s left eye BCVA remained at 20/20, with no symptoms other than a paracentral scotoma. Her PASP remained stable at 45 mmHg with ongoing Bosentan treatment.

## Discussion

Cilioretinal arteries are congenital vascular variants found in approximately one-third of normal eyes, originating from the peripapillary choroid or short posterior ciliary arteries. Their presence can be identified through clinical examination and FFA in about 20–32% of individuals (3). Unlike the central retinal artery, cilioretinal arteries lack autoregulatory mechanisms, making them more susceptible to hemodynamic changes and ischemic events (4).

CilRAO is uncommon, comprising approximately 5% of all retinal artery occlusions. It can be categorized into three primary etiological groups: isolated CilRAO, CilRAO associated with CRVO, and CilRAO attributed to systemic autoimmune conditions such as giant cell arteritis (5–7). In this case, FFA revealed CRVO with delayed and sluggish flow in the cilioretinal artery rather than complete non-perfusion. Additionally, delayed background fluorescence suggested that the CilRAO was caused by hemodynamic disturbances—a high-resistance type of CilRAO associated with CRVO.

The increased intraluminal pressure from CRVO likely led to secondary functional blockage of the cilioretinal artery due to its inability to autoregulate under elevated pressure conditions. The resulting hypoperfusion causes ischemia, particularly in the retinal tissue supplied by the cilioretinal artery. This pathophysiological mechanism aligns with literature suggesting that CRVO-induced elevated capillary pressure may lead to secondary arterial occlusions such as CilRAO (2, 4, 5, 7, 8).

PAH is defined as a persistent pulmonary artery pressure exceeding 25 mmHg at rest or 30 mmHg during exercise (1). The pulmonary arterial (PA) tree, with its fractal branching structure, is designed to evenly distribute blood flow to the alveoli (1). However, conditions such as ventricular septal defect (VSD) that increase pulmonary blood flow can trigger neointimal remodeling, involving intimal hyperplasia, elastic lamina degradation, pericyte infiltration into the intima, and encroachment of smooth muscle cells into the lumen. This remodeling is driven by an imbalance between cell proliferation and apoptosis, upregulation of anti-apoptotic signaling, and persistent inflammation, ultimately causing thickening and fibrosis of the pulmonary artery walls, reduced luminal diameter, and increased vascular resistance (9–11).

While surgical repair of VSD may immediately correct abnormal blood flow, the pre-existing vascular remodeling often persists due to prior exposure to prolonged shear stress and turbulent flow, maintaining elevated pulmonary artery pressures and contributing to ongoing PAH (10, 11). The timing of VSD repair is crucial, as delays can result in irreversible vascular remodeling and permanent pulmonary hypertension (1, 12). As to this case, late surgical correction likely contributed to significant vascular changes and sustained pulmonary hypertension, despite structural defect resolution (13).

PAH can elevate pressure in the superior vena cava, internal jugular vein, cavernous sinus, and ultimately the ophthalmic veins. The elevated pulmonary artery pressure leads to retrograde pressure affecting the right ventricle, right atrium, superior vena cava, and cavernous sinus, disrupting venous outflow from the eye and resulting in CRVO (14). In this patient, echocardiography revealed signs of severe PAH, including right ventricular and atrial enlargement, tricuspid regurgitation, and elevated pulmonary artery pressures, confirming the systemic contribution to the observed CRVO.

The management of this case emphasizes the importance of treating the primary disease, PAH, to alleviate its secondary ocular complications. Bosentan, an endothelin receptor antagonist, plays a pivotal role in lowering pulmonary artery pressure by blocking endothelin-1 (ET-1) receptors on vascular smooth muscle cells, leading to vasodilation and decreased vascular resistance. This reduction in pulmonary artery pressure decreases the retrograde venous pressure transmitted to the ophthalmic veins, thereby alleviating CRVO. By improving pulmonary hemodynamics, Bosentan reduces the venous congestion in the retinal circulation, facilitating the restoration of normal venous outflow. Furthermore, the normalization of retinal venous pressure helps reverse the hemodynamic imbalance causing the CilRAO. Since the CilRAO in this case is hemodynamically induced rather than due to an embolic event, lowering the venous pressure allows for improved perfusion in the cilioretinal artery territory. Although the patient’s central vision may improve with the resolution of CRVO and the reversal of CilRAO, ischemic damage caused by the initial hypoperfusion may leave lasting effects, such as paracentral scotomas.

For elderly patients with combined CilRAO and CRVO, atherosclerosis, thrombophilia, vasculitis, and autoimmune conditions should be assessed. In younger patients, rarer etiologies, including elevated homocysteine levels, syphilis, patent foramen ovale, and HELLP syndrome, must be considered (4). In this case, detailed patient history revealed a past VSD repair, underscoring the need to thoroughly investigate medical history in such instances.

This case represents the first reported instance of CRVO combined with CilRAO and PAMM as initial manifestations of PAH. It underscores the interplay between systemic vascular diseases and ocular health and emphasizes the importance of addressing systemic causes to achieve favorable ophthalmic outcomes.

## Conclusion

The case we presented here underscores the intricate relationship between systemic cardiovascular conditions and ocular health, highlighting the first reported instance of CRVO combined with CilRAO secondary to PAH. Managing such cases emphasizes the importance of addressing the primary disease, PAH, to alleviate secondary ocular complications and prevent severe systemic issues. Bosentan, an endothelin receptor antagonist, played a pivotal role in lowering pulmonary artery pressure, reducing venous pressure, and alleviating CRVO. This approach addresses the root cause of increased retinal venous pressure, providing a comprehensive treatment strategy. Early intervention and holistic treatment are crucial in preventing permanent vision damage and serious systemic complications. This case highlights the need for clinicians to remain vigilant about systemic conditions that can impact ocular health and to adopt a multidisciplinary approach in managing complex cases.

## Data Availability

The original contributions presented in the study are included in the article/supplementary material, further inquiries can be directed to the corresponding author.
